# Long-term social memory of mate copying in *Drosophila melanogaster* is localized in mushroom bodies

**DOI:** 10.1038/s41598-025-88535-x

**Published:** 2025-02-12

**Authors:** Sabine Nöbel, Etienne Danchin, Guillaume Isabel

**Affiliations:** 1 Department of Zoology, Animal Ecology, Martin-Luther-University Halle-Wittenberg, Halle (Saale), Germany; 2 Université Toulouse 1 Capitole and Institute for Advanced Study in Toulouse (IAST), Toulouse, France; 3 Laboratoire Évolution & Diversité Biologique (EDB UMR 5174), Université de Toulouse Midi-Pyrénées, CNRS, IRD, UPS. 118 route de Narbonne, 31062 Toulouse, France; 4 Centre de Recherches sur la Cognition Animale (CRCA), Centre de Biologie Intégrative (CBI), CNRS UMR 5169, Université de Toulouse Midi-Pyrénées, Toulouse, France

**Keywords:** *Drosophila melanogaster*, Kenyon cells, Long-term memory, Mushroom bodies, Social learning, Cultural evolution, Sexual selection, Long-term memory, Learning and memory, Sexual behaviour

## Abstract

Long-term social memory (LTSM) is a key feature to elicit the cultural inheritance of behaviour independently of genetics. However, the neurobiological basis of LTSM remains largely unknown. We previously used the *Drosophila* animal model, which is known to perform mate copying through observational learning of the mate choice of conspecifics to show that the expression of the *rutabaga* gene, a calcium/calmodulin-dependent adenylyl cyclase (AC-Rut+) that acts as a coincidence detector enabling associative learning, is necessary and sufficient in the γ-Kenyon cells (KCs) of the mushroom bodies (MBs). Here, we show that the expression of AC-Rut^+^ in both the γ- and the α/β-KCs is required for LTSM involving de novo protein synthesis in a mate-copying context, whether using demonstrations involving real flies or involving pictures of copulating conspecifics. Thus, pathways of short- and long-term memory show considerable overlap in the MBs across social vs. asocial learning contexts.

**Supplementary Information:**

The online version contains supplementary material available at 10.1038/s41598-025-88535-x.

## Introduction

Social learning, which refers to the capacity to learn from other individuals, is central to the transmission of information among individuals within a population, provided that the information is memorized over sufficient time to be copied by other individuals. Furthermore, when coupled with the ability to learn information from the majority of individuals in a population (i.e. conformity^1–10^), long-term memory of socially learnt traits can lead to the intergenerational transmission of specific traits and habits hence leading to a new form of inheritance, namely cultural inheritance, which in turn can foster the emergence of local cultural traditions that can potentially persist over many generations^11,12^. It is thus crucial to study the long-term memorization of socially learnt traits, which we here call long-term social memory (LTSM). Although such LTSM has been described in a few animal species^12–15^, little is known about its neurobiological basis.

Here, using the mate-copying paradigm, we investigate this question in an invertebrate, *Drosophila melanogaster*, which has all the cognitive abilities to perform cultural traditions. Mate copying is a form of social learning in which, after observing another demonstrator females’ mate choice, an observer female preferentially mates with the same male (“individual based” mate copying) or with males of the same phenotype (“trait based” mate copying) as the one chosen during the demonstration^16,17^. This strategy is widespread in the animal kingdom from humans to fruit flies (reviewed in^18^).

Typically, in the fruit fly *D. melanogaster* mate-copying experiments have two phases, a demonstration during which a virgin observer female can watch another female copulating with a male of a certain phenotype at the expense of another male with a contrasting phenotype that is thus being rejected, followed by a mate-choice test during which the observer female is given the choice between two males, one of each phenotype^19^. The characteristics of mate copying in *Drosophila* have been shown to potentially lead to local traditions of preferring a certain male phenotype over another under several key conditions, one of which being the long-term social memory (LTSM) of the learned preference^12^, therefore raising the question of the mechanism of LTSM. In effect, building LTSM of observed mate choices greatly enhances the likelihood that a preference for a specific male phenotype can be copied, hence potentially invading a local population and leading to the cultural inheritance of sexual preferences^12^.

Mammals are known to build long-term social memory (LTSM) as they recognize familiar, non-kin individuals after a long time, sometimes even decades. This LTSM seems to be located in the hippocampus and it was assumed that a good social memory is beneficial especially in fluid social systems^13–15^. In *Drosophila*, the sensory modalities required for mate copying appear to be very basic and robust because it has been shown recently that even showing a simple photo of a copulation is sufficient to emulate mate copying^20,21^, demonstrating that visual cues alone are sufficient to elicit mate copying in the fruit fly. Two brain structures known to govern discriminative visual learning are candidates for promoting discriminative visual social learning: the central complex, whose activity is necessary for Skinnerian operant learning^22^, and the mushroom bodies (MBs) required for classical Pavlovian learning^23,24^. A previous study has demonstrated the importance of the MBs for the acquisition and/or short-term memory (STM) of the information provided during demonstrations in mate-copying experiments^21^. Interestingly, the MBS are comparable to the human hippocampus in term of its function in learning and memory. After viewing spaced consistent repeated demonstrations of mating conspecifics, female fruit flies memorise the locally preferred male phenotype over the long term, a process that requires de novo protein synthesis^12^, as in associative classical olfactory LTM^25–28^.

There is already evidence that some of the Kenyon cells (KCs) of the MBs are involved in associative learning, as demonstrated by studies on mutated genes such as *rutabaga*^29–34^ and other genes, as well as by investigations into the role of neurons involved in this process (for review^35,36^). Interestingly, the central complex but not KCs is required in operant visual learning^22,37^. However, since the expression of the *rutabaga* gene is necessary in the MBs for the STM of mate copying, it is very likely that they are also involved in LTSM in one way or another, as this is the case in other learning and memory processes described in *Drosophila*. However, the specific neurons where such gene expression is required in visual LTSM are unknown.

The MBs consist of roughly 4000 Kenyon cells (KCs) that form three distinct lobes in each hemisphere, the α/β-, α’/β’- and γ-lobes^38,39^. It has been demonstrated that the α’/β’-KCs outputs are essential for both olfactory appetitive and aversive learning and memory consolidation processes^40,41^. Regarding the neurobiological localization of LTM processes, mutants with structural defects of the α-lobes cannot form olfactory LTM^42,43^. Interestingly, there is also evidence that γ-lobes can be involved in the establishment of olfactory LTM traces distinct from those formed in the α/β-lobes^44,45^.

The question of the localization of the association between the two stimuli necessary for the formation of associative LTSM can be efficiently addressed by studying a mutant with a deficiency in Rutabaga expression. This protein is known to be crucial for neuronal plasticity. The Rutabaga protein (AC-Rut^+^) is indeed a Ca_2_^+^-Calmodulin adenylyl-cyclase that functions as a coincidence detector between the olfactory stimulus and the sugar/electroshock stimulus^33,34,46,47^. The nature of this enzyme in conjunction with in vivo imaging experiments^48,49^, showed that this protein is synergistically activated by two neurotransmitters involved in the simultaneous stimulation of the olfactory pathway on one hand, and the electroshock or appetitive pathway on the other hand.

Here, using the *rutabaga* rescue approach that already proved to be efficient in the study of mate-copying learning and STM^21^, we identify the neurons in which the expression of a functional Rutabaga protein is sufficient to re-establish a functional LTSM in a mate-copying context. We use an already established wild type *rutabaga + cDNA* construct under control of different Gal4 drivers to spatially restrict *rutabaga +* expression in an otherwise *rutabaga* lacking mutant female to figure out in which sets of neurons in MBs is the expression of a functional AC-Rut^+^ protein necessary to rescue mate copying. In all the mate-copying experiments in this paper, the demonstrations involved a series of five spaced training sessions in which *Drosophila* females could observe and then copy the choice of artificially dusted green and pink males^12^. To figure out what MB lobes are necessary for this type of LTSM, we used observer females bearing different genetic constructs. We crossed *rut*^*2080*^;UAS-*rut*^+^ females with males from different Gal4 lines to determine which substructure of the MBs is involved. Females lacking either UAS-*rut*^ +^ or lacking Gal4 were used as control groups. In addition to demonstrations with real flies, we also showed photos of copulating flies during the demonstration to support the results of our experiment with real flies in the demonstrations. Whatever the demonstration method (live vs. photos), we found that the functional expression of the AC-Rut^+^ is necessary in both the α/β- and γ-KCs of the MBs to rescue long-term social memory of mate copying.

## Results

### Long-term social memory for mate copying requires the expression of the coincidence detector Rutabaga (AC-Rut^+^) in α/β- and γ-Kenyon cells

**Fig. 1:**
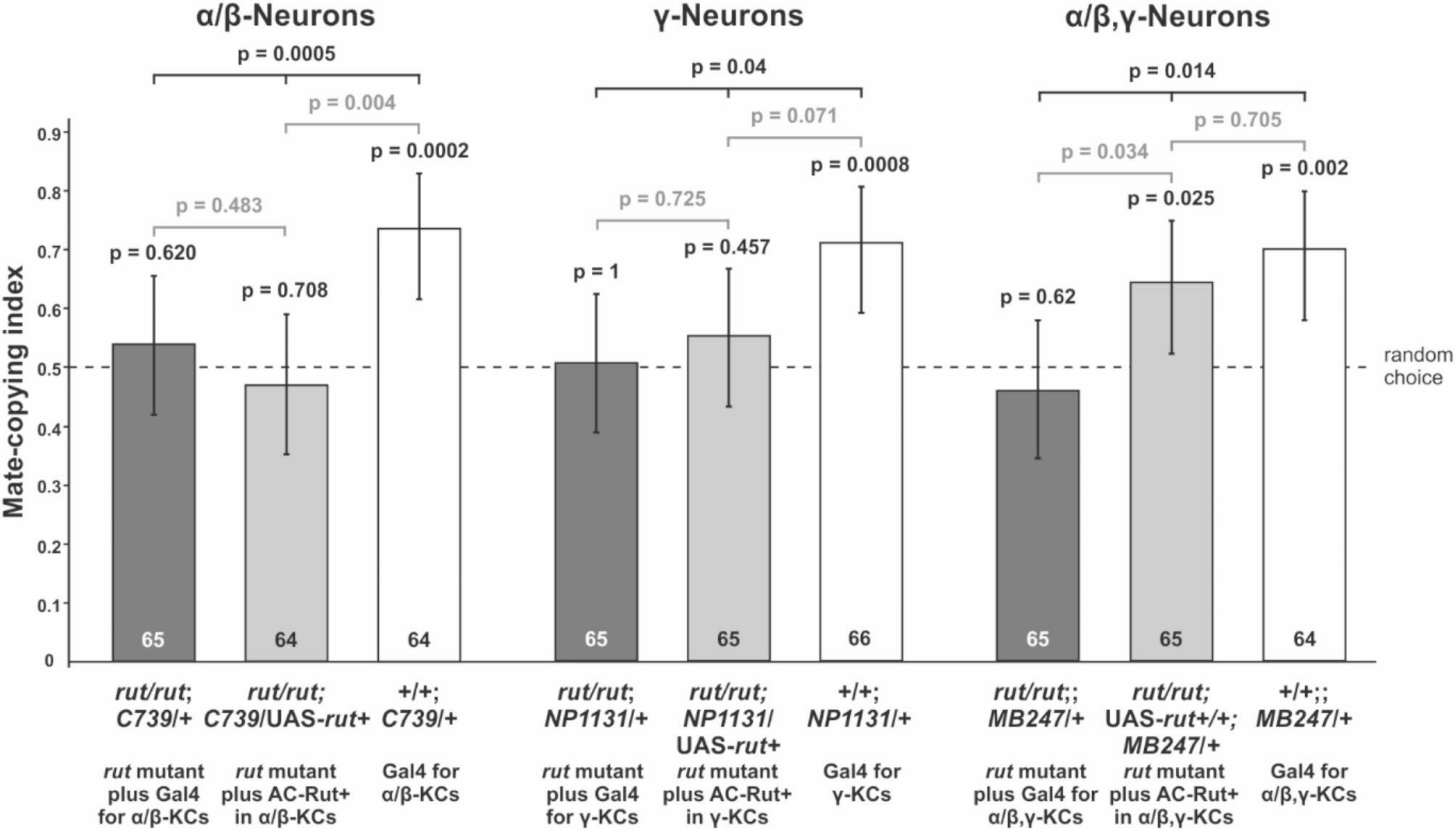
The Rut-AC ^+^ is required in both the α/β- and γ-KCs to induce LTSM of mate copying. Although the rut/rut observer females we used in these experiments were not able to express a functional adenylyl-cyclase coincidence detector, some of the various constructs we used allowed us to re-establish the expression of the wild type *rutabaga* gene in very specific neuron subsets of the MB, namely the α/β-Kenyon cells (using the Gal4 driver line *C739*), the γ-Kenyon cells (using the Gal4 driver line *NP1131*) or both (using the Gal4 driver line *MB247*). Only the use of the latter UAS/Gal4 fully rescued mate copying. Statistics: above bars, P-values of the binomial tests of departure from random choice (represented by the dashed line), and that above the black horizontal bar is that of the treatment effect (GLMM). Grey bars represent post-hoc Fisher tests and error bars represent Agresti-Coull 95% confidence intervals.

First, to express AC-Rut^+^ in the *rut* mutant context in the α/β-KCs, we used the Gal4 driver line *C739* (three left bars of Fig. 1). Although such *rut/rut*;*C739*/UAS-*rut*^+^ observer females are able to express a functional Rutabaga protein (AC-Rut^+^) in the α/β-KCs, they chose randomly between the two differently coloured males (binomial test: *n* = 64, *P* = 0.708; second bar in Fig. 1), as did the control treatment that were homozygous for the *rut* mutation and carried only the Gal4 driver *C739* (*rut/rut*;*C739*/+; binomial test: *n* = 65, *P* = 0.620, first bar Fig. 1). Contrastingly, wild type *rut*^*+*^ females that carried only a hemizygote copy of *C739* showed normal copying behaviour (binomial test: *n* = 64, *P* = 0.0002, third bar Fig. 1). When comparing all three treatments we found that the treatment effect was significant (GLMM: Χ^2^ = 12.073, df = 2, *P* = 0.0005). The observer females lacking (*rut/rut*;*C739*/+) or expressing AC-Rut^+^ (*rut/rut*;*C739*/UAS-*rut*^+^) do not significantly differ from each other (post hoc Fisher test: *n* = 129, *P* = 0.483). Females expressing AC-Rut^+^ (*rut/rut*;*C739*/UAS-*rut*^+^) differ significantly from the control group carrying the Gal4 driver *C739* only (post-hoc Fisher test: *n* = 128, *P* = 0.004). Thus, AC-Rut^+^ expression only in α/β KCs is not sufficient to perform mate copying and achieve LTSM.

To address the AC-Rut^+^ involvement in γ-KCs, we used the Gal4 driver line *NP1131* (middle group of Fig. 1). We could not rescue mate-copying behaviour in *rut* observer females with a functional AC-Rut^+^ copy only in the γ-KCs (*rut/rut*;UAS-*rut*^+^/NP1131; binomial test: *n* = 65, *P* = 0.457; fifth bar in Fig. 1). The control group of *NP1131*/+ females, that are not mutant for AC-Rut^+^, mated significantly more often with the male colour observed in the demonstration (binomial test: *n* = 66, *P* = 0.0008; sixth bar in Fig. 1), while observer females with an inactive AC-Rut^+^ (*rut/rut*;*NP1131*/+) in the γ-KCs chose randomly (binomial test: *n* = 65, *P* = 1.0; fourth bar in Fig. 1). When comparing all three treatments concerning the γ-KCs we found that the treatment effect was significant (GLMM: Χ^2^ = 6.448, df = 2, *P* = 0.04). Again observer females lacking (*rut/rut*;*NP1131*/+) or expressing AC-Rut^+^ (*rut/rut*;*NP1131*/UAS-*rut*^+^) do not significantly differ from each other (post-hoc Fisher test: *n* = 130, *P* = 0.725). However, females expressing AC-Rut^+^ (*rut/rut*;*NP1131*/UAS-*rut*^+^) do not significantly differ from the control group carrying the Gal4 driver *NP1131* only (post-hoc Fisher test: *n* = 131, *P* = 0.071). Thus, contrary to what we observed during our study on learning and STM^21^, AC-Rut^+^ in γ-KC is not sufficient to elicit LTSM.

In the next step, we expressed AC-Rut^+^ in both, the α/β- and γ-KCs with the Gal4 driver *MB247* (right group of Fig. 1). This time we could rescue mate copying in observer females expressing AC-Rut^+^ (*rut/rut*;UAS-*rut*^+^/+;*MB247*/+; binomial test: *n* = 65, *P* = 0.025; eight bar in Fig. 1) while the control groups behaved as expected. The wild type *rut*^+^ observer females carrying only the Gal4 driver (*MB247*/+) copied the choice of the demonstrators (binomial test: *n* = 64, *P* = 0.002; ninth bar in Fig. 1), while females lacking a functional AC-Rut^+^ (*rut/rut*; *MB247*/+) chose randomly (binomial test: *n* = 65, *P* = 0.620; seventh bar in Fig. 1). When comparing all three treatments we found that the treatment effect was significant (GLMM: Χ^2^ = 8.5458, df = 2, *P* = 0.014). Here observer females lacking (*rut/rut*;;*MB247*/+) or expressing AC-Rut^+^ (*rut/rut*;UAS-*rut*^+^/+;*MB24*7/+) significantly differ from each other (post-hoc Fisher test: *n* = 130, *P* = 0.034). Females expressing AC-Rut^+^ (*rut/rut*;UAS-*rut*^+^/+;*MB24*7/+) did not significantly differ from the control group carrying the Gal4 driver *MB247* only (post-hoc Fisher test: *n* = 129, *P* = 0.705). Hence, LTSM of mate copying requires the expression of AC-Rut^+^ in both α/β- and γ-KCs.

### Long-term social memory pathways are similar when using live versus picture demonstrations

**Fig. 2:**
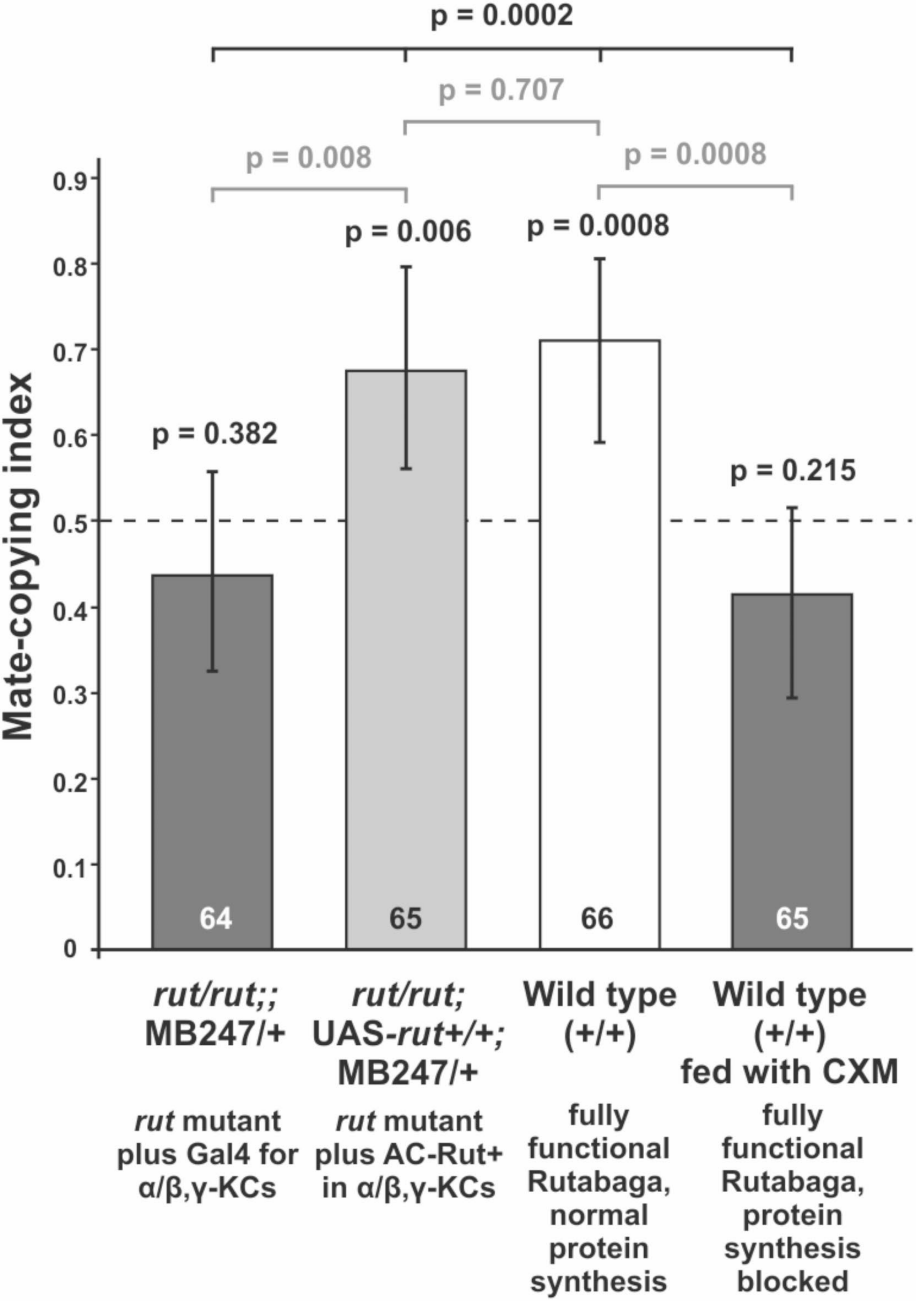
Establishment of LTSM in mate-copying experiments using photos in the demonstration. We expressed the wild type AC-Rut^+^ gene in the α/β- and γ-KCs of the MB with the Gal4 driver line *MB247* and used pictures of copulating flies instead of live demonstrations. Wild type (third bar) and wild type females fed with the protein synthesis blocker CXM (fourth bar) were used as control. Statistics: above bars, P-values of the binomial tests of departure from random choice (represented by the dashed line), and that above the horizontal bar is that of the treatment effect (GLMM) including post-hoc tests in grey. Error bars represent Agresti-Coull 95% confidence intervals.

As a proof of concept, we repeated the experiments above, replacing the five live demonstrations with five photos of copulating flies (all of the same colour). We found that WT flies copied the choice shown on the photos (binomial test: *n* = 66, *P* = 0.0008; third bar in Fig. 2). We also found that observer females with a functional AC-Rut^+^ copy in both α/β- and γ-KCs built a full LTSM (*rut/rut*;UAS-*rut*^+^/+;*MB247*/+; binomial test: *n* = 65, *P* = 0.006; second bar in Fig. 2). Contrastingly, *rut/rut*;;*MB247*/+ females lacking AC-Rut^+^ did not show LTSM in mate copying (binomial test: *n* = 64, *P* = 0.382; first bar in Fig. 2). Similarly, wild type females fed with the protein synthesis blocker cycloheximide (CXM) prior to experiments chose randomly between males (binomial test: *n* = 65, *P* = 0.215; fourth bar in Fig. 2). There was a significant difference in mate-copying scores between the different treatments (GLMM: Χ^2^ = 19.8341, df = 3, *P* = 0.0002, Fig. 2), while the picture ID had no influence (*P* = 0.807). Here observer females lacking (*rut/rut*; *MB247*/+) or expressing AC-Rut^+^ (*rut/rut*;UAS-*rut*^+^/+;*MB24*7/+) significantly differ from each other (post-hoc Fisher test: *n* = 129, *P* = 0.008). Females expressing AC-Rut^+^ (*rut/rut*;UAS-*rut*^+^/+;*MB24*7/+) do not significantly differ from the wildtype control group (post-hoc Fisher test: *n* = 131, *P* = 0.707). Wild type female fed with the protein synthesis blocker CXM differ significantly from normal wild type females (post-hoc Fisher test: *n* = 131, *P* = 0.0008). This last result shows that the establishment of a LTSM also involves de novo protein synthesis when photo demonstrations are used, in a manner similar to that observed in a previous study using live demonstrations^12^.

Altogether, these results provide compelling evidence that the association between the US and CS stimuli necessary to establish LTM of visual social learning in a mate-choice context occurs in the α/β- and γ-KCs.

## Discussion

Our aim was to identify the specific MB lobes necessary for LTSM of mate copying. We found that the expression of AC-Rut^+^ is required in both the γ- and the α/β-KCs for LTSM involving de novo protein synthesis, regardless of whether the demonstrations used involved real flies or pictures of copulating conspecifics. Therefore, the pathways involved in short^21^ and long-term memory exhibit significant overlap in the MBs across both social and asocial learning contexts.

Concerning methodology, as the spaced training with five consistent demonstrations is very demanding in terms of delicate experimental manipulations, using pictures for the demonstrations instead of real flies greatly facilitates replication for the deciphering of the neurobiology of LTSM in the context of mate choice. Furthermore, it significantly reduces the number of flies used for each experiment (3 instead of 18) hence diminishing needs for fly maintenance and other manipulations. In the future the demonstration phase could be even automatized and support the reproducibility of experiments by enabling full control over stimuli.

The aim of the present study is to provide first insights into the neurological mechanisms of long-term memory in mate copying as an example of social learning and to compare it with what is known about the mechanisms of acquisition and memorisation in a short-term context. We previously provided similar insights on the neuronal pathway of mate copying and found that the expression of the AC-Rut^+^ coincidence factor in the γ-KCs of the MBs is necessary and sufficient in the acquisition phase in a STM context^21^. That previous study already showed that this form of discriminative social learning requires the same KCs as non-social Pavlovian learning, suggesting that pathways of social and asocial learning overlap significantly^33^. In the current study, we found that in LTSM context we need the presence of the coincidence detector AC-Rut^+^ in both, the α/β- *and* γ-KCs, to rescue the mate-copying behaviour at 24 h. This suggests that there is no difference between social and non-social aversive (with electric shocks) LTM as shown by Blum and colleagues^29^ who, using the *rutabaga* gene, found that α/β- and γ-neurons are necessary to build a full non-social aversive LTM. However, our results differ from those of Trannoy and colleagues^31^ in an appetitive (sugar reward) context who found that AC-Rut^+^ expression in only α/β-neurons are sufficient for generating appetitive LTM. Our result is in so far surprising as we showed previously that watching a copulation is more rewarding than aversive, especially as females later choose to copulate with the same phenotype as the demonstrators and not to avoid those males^50^. Further studies are needed to decipher the differences between non-social olfactory and social visual LTM, whether these LTMs are aversive or appetitive.

In addition to finding the structures involved in the LTSM of mate copying, we were able to replicate twice the results of Danchin and colleagues^12^, once with live and once with photo demonstrations, showing that *Drosophila* females are able to establish a long-term social memory of a mate-choice preference they observed, over at least 24 h, and that this establishment involves de novo protein synthesis, using pathways that involve the same neurons expressing AC-Rut^+^ in these two experimental conditions (photo vs. live). We show that the coincidence detector AC-Rut^+^ is necessary in the MBs and plays an important role in the establishment of both short-term and long-term social memory in a mate-choice context. We suspect that multiple repeated demonstrations could potentiate AC-Rut^+^ activation and favour long-term consolidation by activating the Protein-Kinase A and subsequently recruiting transcription factors that lead to de novo protein synthesis. AC-Rut^+^ expression is not sufficient in α/β-neurons to generate LTSM processes, suggesting a γ-KCs role to maintain AC-Rut^+^-dependent LTSM in α/β-neurons.

A review of the current knowledge about the role of the *rutabaga* gene in learning and memory formation in the fruit fly shows that different lobes of the MBs can be involved according to the context, whether social or asocial, appetitive or aversive, visual or olfactory in both short and long-term memory. However, the main message of this review is that there is considerable overlap in the pathways of memory formation in these different contexts, which makes sense as it would be surprising that natural selection has favoured completely different pathways for each specific context.

Based on this knowledge, it will now be possible to trace the pathways of learning in general, both upstream and downstream, and thus, gain insight into how information is learned and memorised, and the extent to which the cognitive pathways differ between different learning contexts. Our expectation is that learning in different contexts will share a common generic pathway, with differences occurring only upstream or downstream of this common pathway depending on the sensory modalities involved and the type of response produced. We are particularly interested in the origin of social learning as the main mechanism of cultural inheritance, because the importance of LTSM for the evolution of social learning and the maintenance of a large repertoire of cultural variants in a population with changing environments has just been demonstrated in a theoretical study^51^.

## Methods

### Fly maintenance

We used the common laboratory strain Canton-S of *D. melanogaster* (later we refer to it as wild type (WT)) and the mutant lines *rut*^*2080*^, *rut*^*2080*^;UAS-*rut*^+^, and the three following Gal4 lines *C739*, *NP1131*, and *MB247*. The Gal4 lines were outcrossed for at least five generations to *w*^*1118*^ flies with Canton-S background before experiments started.

All flies lines were raised in 30 ml vials containing 8 ml corn meal-agar-yeast medium at 25 °C and ~ 60% humidity with a 12:12 h light: dark cycle. Flies were sexed and sorted without anaesthesia by gentle aspiration within 2–6 h after emergence and kept in unisex groups of 7 females or 15 males per vial before experiments. Experimental flies were virgin and three or four days old. Between demonstration and test all females were kept in individual tubes.

Experiments were conducted under the same conditions as breeding (12 h daylight, 25 °C, ~ 60% humidity). Observer females were of different genotypes, while demonstrators and test males were always from the Canton-S strain. We created two artificial male phenotypes by randomly dusting males with green or pink powders^52^, which created two contrasting phenotypes independent of any genetic variation. All males and females were used only once, which means that males used in the mate-choice test always differed from those used in the demonstration. All fly manipulations were performed by gentle aspiration without anaesthesia.

### Experimental protocol

**Fig. 3:**
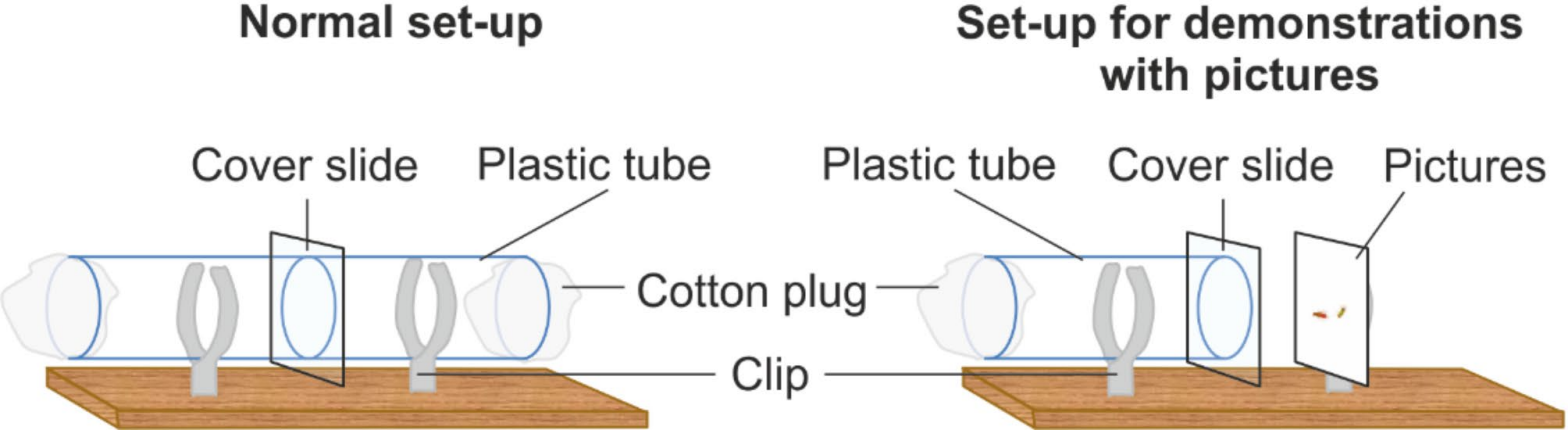
Schematic drawing of the experimental set-ups used. On the left you can see the normal set-up with live demonstrations and mate-choice tests of the experiments with pictures. The modified set-up for demonstrations with pictures instead of living flies is on the right.

**Fig. 4:**
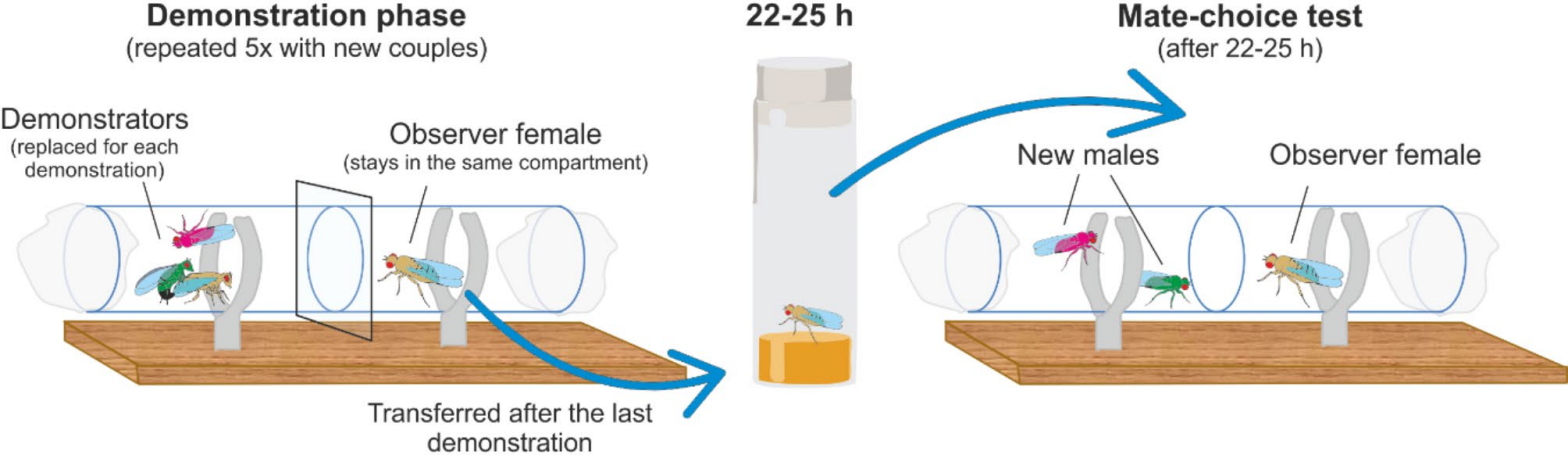
Schematic drawing of the experimental protocol with live demonstrators. The demonstration phase consisted of five successive observation phases with already mating couples of the same colour. The observer female remains in the same compartment of the whole demonstration phase, only demonstrators were replaced After the last demonstration, the observer female was transferred to a food vial for 22–25 h and tested afterwards with two new males.

Experiments took place in double plastic tubes (1.1 cm × 3 cm each) separated by a microscopy cover slide (16 mm × 16 mm, Fig. 3). Typically, each mate-copying experiment had two phases: a demonstration followed by a mate-choice test. Demonstrations consisted in a single virgin demonstrator female placed with two virgin males, one of each colour, for 30 min on one side of the tubes, and a naïve, virgin observer female on the other side of the tubes separated by the glass partition. The copulation of the demonstrator female with one of the coloured males provided positive information for that male colour and negative information for the other male phenotype. As copulation lasts approx. 20 min in *D. melanogaster*, the observer female had plenty of time to gather information about the mate choice of the demonstrator female. However, to elicit long-term memory of the observed mate choice it is necessary to perform a spaced training^12^. That is, we used the classical speed learning protocol^19^ and instead of a single demonstration, we presented a series of five consecutive demonstrations, in each of which the demonstrator female copulated with the same-coloured male phenotype, with the other male phenotype being rejected, with 15–30 min breaks between demonstrations. The observer female was placed in one of the tubes at the beginning of the experiment and remained there for the whole demonstration phase to minimize stress due to handling. As in previous studies^12,19^, we used already copulating pairs for the demonstration to ensure that observer females always saw the same male phenotype mating, i.e., received consistent information about which male phenotype is preferred. To create copulating pairs, we put virgin females with males of the desired colour in an extra tube and transferred them by gentle aspiration shortly after they started copulating. As soon as the demonstrators parted, they were removed from the tube marking the beginning of the break before the next demonstration. After the fifth and final demonstration, each observer female was transferred into an individual food tube and remained until the test 22–25 h later (Fig. 4).

For the mate-choice test, we first inserted the observer female in one side of the tube set-up and then a new pair of males, one of each phenotype, in the demonstration side of the tube. Next, we removed the partition so that the observer female could make her own choice within the next 30 min. To control for male competition that can never be excluded in free ranging individuals, we recorded whether both males courted the female. For the analyses we only kept replicates in which both males actively courted the female (wing flapping), as this was the only situation when we were sure that the observer females were in a real situation of choice. All replicates were run as blocks of 6 trials with cardboard separations between experimental set-ups to prevent information exchange between the flies and prevent disturbance by the surrounding.

Replicates, where the observer female copulated with the male of the phenotype preferred during the demonstration (copied), were attributed a mate-copying score of 1, versus 0 in the opposite case. The mate-copying index (MCI) is the mean mate-copying score for each treatment, which corresponds to the proportion of females copulating with the male of the same phenotype as the one that was apparently selected by the demonstrator female. Mate-copying index around 0.5 indicated random choice by observer females, while values above 0.5 revealed mate copying.

As said before, for the analysis we took only replicates that fulfilled a minimum criterion of quality, which are (i) whether copulation occurred during the mate-choice test and (ii) if both males courted the observer female before copulation. Other situations were discarded from the analysis. We tested in total 1644 observer females and discarded 1061 replicates where demonstration failed, only one male courted the female or no copulation was observed within the 30 min mate-choice test.

### Treatments

With some residual Gal4 expression^53^, Gal4 *C739*, Gal4 *NP1131* and Gal4 *MB247* are drivers widely used to assess their implication in α/β-KCs, γ-KCs and α/β-γ KCs respectively^29,40,53–60^. We crossed these lines with *rut*;UAS-*rut*^+^ or wild type flies. If one of these lobes is involved in mate copying, we expected to rescue the behaviour only when observer females were *rut*^2080^ hemizygous, UAS-*rut*^+^ heterozygous and Gal4 heterozygous, while females carrying only the Gal4 should choose randomly (for crossing schemes see the supplements).

If we find that one or a combination of these MB lobes is involved in mate copying with live demonstrations, we use the same line and test it with photos during the demonstration to confirm our results. In addition, we test if this form of LTSM depends on de novo protein synthesis by feeding a control group of wild type flies 20–25 h before the demonstrations with a 35 mM cycloheximide (CXM) sucrose solution. CXM inhibits protein synthesis and if LTSM depends on de novo protein synthesis after the demonstrations these flies should mate randomly.

### Proof of concept using photos during the demonstration

In addition to the demonstrations with real flies, we repeated the same experiments, replacing live demonstrations of real copulations with mere photos of copulating flies (Fig. 4). We used the same pictures as a previous study^20^. On each photo, a couple plus a rejected male of the opposite colour were presented on white background either in top or front view. Angles and position of flies varied between pictures. The size of the flies on the printed photo (printed on photo paper) was about 2.5 mm, which corresponds to their natural size. The only manipulations made to the pictures were to remove the background and enhance the green and pink dusting of the males. From a pool of 25 pictures, we created booklets with photos of 5 different copulating pairs plus rejected male so that observer females saw 5 different copulations—with demonstrator females always copulating with males of the same colour. Each booklet showed the pictures in a different order and included a mixture of top and lateral views of the copulating pairs. Each photo was shown for 25 min and covered with cardboard during the 15 min breaks. After the 15-min break, we removed the cardboard cover and turned the page to reveal the next photo, which marked the beginning of the next demonstration in the series. After the fifth and last demonstration, each observer female was transferred to an individual food tube where they remained for 22–25 h when the mate-choice test was performed. We tested in total 540 observer females and discarded 280 replicates where only one male courted the female or no copulation was observed within the 30 min mate-choice test.

### Animal welfare note

Our study involved populations of *D. melanogaster* that have been maintained exclusively under laboratory conditions for hundreds of generations. The current study includes behavioural observations of *D. melanogaster* which required no ethical approval and complied with French laws regarding animal welfare. We handled flies by gentle aspiration without anaesthesia to minimize discomfort. After the experiments, individuals were euthanized in a freezer at −20 °C.

### Statistical analysis

All statistical analyses were performed with the R software (version 4.0.2^61^). The difference from the random choice was tested with a two-tailed binomial test. To test for a treatment effect, mate-copying scores were analysed in a generalized linear mixed model (GLMM) with binary logistic regression (package *lme4*^62^). All models included air pressure as fixed effect as it was shown to influence mate copying in *D. melanogaster*^19^, however, accounting or not for air pressure did not change any conclusion. In experiments using pictures during the demonstration, we also included the photo ID as fixed effect. We also included a random block effect to account for non-independence of flies from the six trials that were run simultaneously. Significance of fixed effects was tested using Wald chi-square tests implemented in the ANOVA function of the *car* package^63^. All starting models included interactions between fixed effects. We applied a backward selection method using P-values, by dropping out non-significant effects, starting with the highest order interaction.

## Electronic supplementary material

Below is the link to the electronic supplementary material.

Supplementary Material 1

Supplementary Material 2

## Data Availability

The datasets generated during the current study are available in the Dryad repository 10.5061/dryad.gxd2547xf.
