# Supplementary material for: Long-term social memory of mate copying in Drosophila melanogaster is localized in mushroom bodies
Source: Sci Rep. 2025 Feb 12;15:5262. doi: 10.1038/s41598-025-88535-x (PMC11822108; doi:10.1038/s41598-025-88535-x)
Supplement: Supplementary file 1 — Supplementary Material 1 [file 41598_2025_88535_MOESM1_ESM.pdf]

Supplementary Material -
Long-term social memory of mate copying in *Drosophila melanogaster* is localized in mushroom bodies

Sabine Nöbel[1,2,3*], Etienne Danchin[3,4] & Guillaume Isabel[4]

[1] Department of Zoology, Animal Ecology, Martin-Luther-University Halle-Wittenberg, Halle (Saale), Germany
[2] Université Toulouse 1 Capitole and Institute for Advanced Study in Toulouse (IAST), Toulouse, France
[3] Laboratoire Évolution & Diversité Biologique (EDB UMR 5174), Université de Toulouse Midi-Pyrénées, CNRS, IRD, UPS. 118 route de Narbonne, F-31062 Toulouse, France
[4] Centre de Recherches sur la Cognition Animale (CRCA), Centre de Biologie Intégrative (CBI), CNRS UMR 5169, Université de Toulouse Midi-Pyrénées, Toulouse, France

*To whom correspondence should be addressed:
Sabine Nöbel
Department of Zoology, Animal Ecology, Martin-Luther-University Halle-Wittenberg, Halle (Saale), Germany
Email: sabine.noebel@zoologie.uni-halle.de

Food recipe

1 l tap water
70 g inactive dry yeast (Dutscher)
70 g organic corn flour (S.A.S. Alisa, FR)
14 g type 1 agar (Dutscher)
10 g Tegosept (Dutscher)
3 g propionic acid (Dutscher)
0.25 g Vanderzant vitamin mixture for insects (Sigma Aldrich)
20 ml absolute ethanol (Fisher)

Powders

The powders used to colour the males were #1162R Luminous Powder-Red and B-731 Lime Green Fluorescent Pigment both purchased from BioQuip Products, Inc.

CXM

To test whether LTM is dependent of *de novo* protein synthesis we feed observer females 20 – 25 h before the demonstration phase with a cycloheximide solution (CXM, Sigma C7698, St Louis, MO, USA). To prepare the CXM solution we added 100 mg of 94% pure CXM to 10 ml of a 5% sucrose solution. 125 µl of this solution was administered on a piece of Whatman filter paper (10 mm x 25 mm) in a 15 ml Falcon tube.

Crossing schemes to create the desired mutant lines

1. Step	2. Step	Genotype to test
rut/rut; CyO/If x<br>*+/y; C739/C739*	*rut/rut; +/+ x*<br>*rut/y; C739/CyO*	*rut/rut; C739/+*
rut/rut; CyO/If x<br>*+/y; C739/C739*	*rut/rut;; UAS-rut*$^+$*/UAS-rut*$^+$ *x*<br>*rut/y; C739/CyO*	*rut/rut; C739/UAS-rut*$^+$
	+/+; +/+ x<br>*+/y; C739/C739*	*+/+; C739/+*
rut/rut; CyO/If x<br>*+/y; NP1131/NP1131*	*rut/rut; +/+ x*<br>*rut/y; NP1131/CyO*	*rut/rut; NP1131/+*
rut/rut; CyO/If x<br>*+/y; NP1131/NP1131*	*rut/rut; UAS-rut*$^+$*/UAS-rut*$^+$ *x*<br>*rut/y; NP1131/CyO*	*rut/rut; NP1131/UAS-rut*$^+$
	+/+; +/+ x<br>*+/y; NP1131/NP1131*	*+/+; +/NP1131*
rut/rut;; e,Δ/TM3,Sb x<br>*+/y;; MB247/MB247*	*rut/rut;; +/+ x*<br>*rut/y;; MB247/TM3,Sb*	*rut/rut;; MB247/+*
rut/rut;; e,Δ/TM3,Sb x<br>*+/y;; MB247/MB247*	*rut/rut; UAS-rut*$^+$*/UAS-rut*$^+$ *x*<br>*rut/y;; MB247/TM3,Sb*	*rut/rut; UAS-rut*$^+$*/+;*<br>*MB247/+*
	+/+;; +/+ x<br>*+/y;; MB247/MB247*	*+/+; MB247/+*
