# Supplementary figures and images for: Long-term social memory of mate copying in Drosophila melanogaster is localized in mushroom bodies
Source: Sci Rep. 2025 Feb 12;15:5262. doi: 10.1038/s41598-025-88535-x (PMC11822108; doi:10.1038/s41598-025-88535-x)

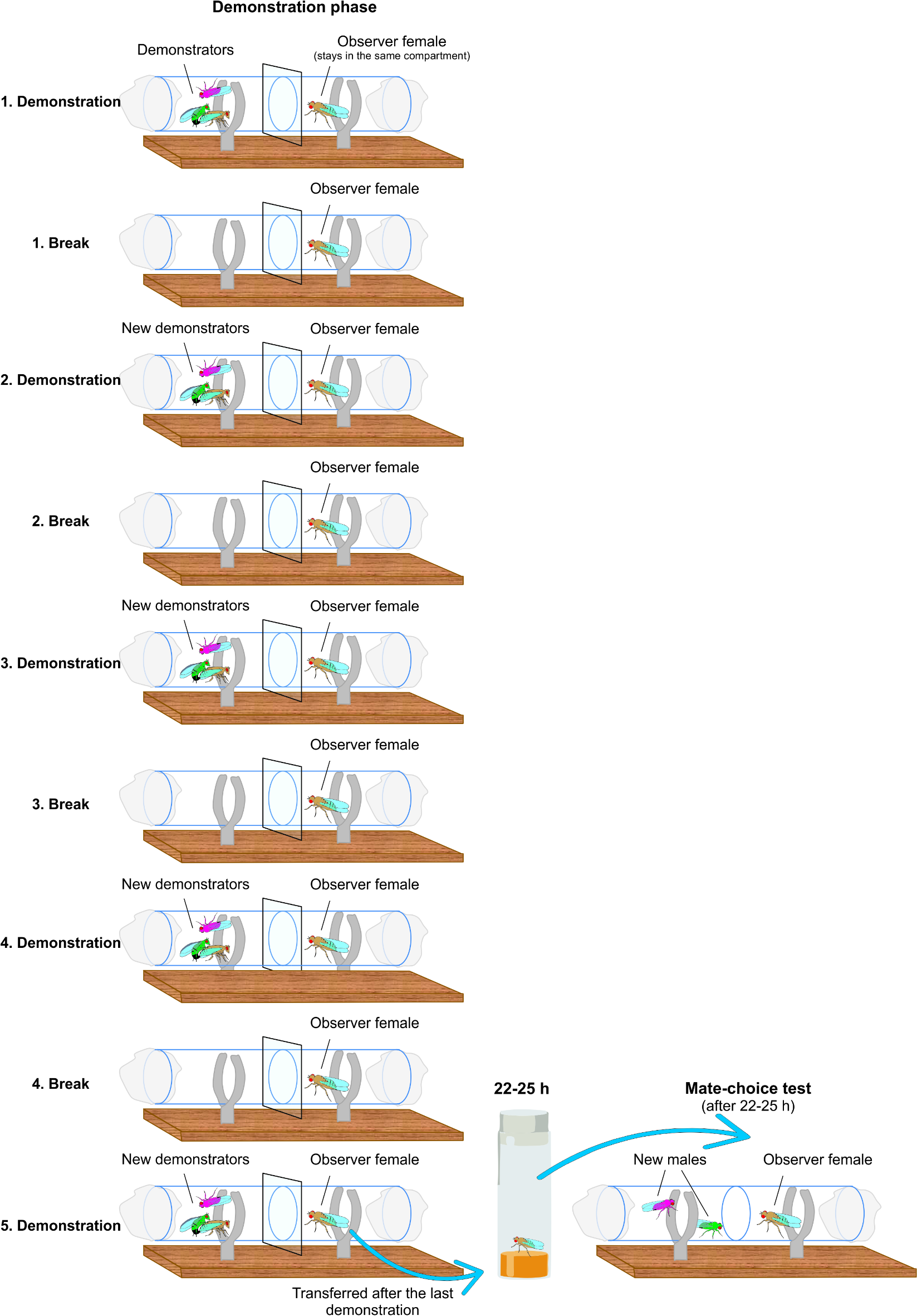

Supplement: Supplementary file 2 — Supplementary Material 2 [file 41598_2025_88535_MOESM2_ESM.png]
